# Referral paths in the U.S. physician network

**DOI:** 10.1007/s41109-018-0081-4

**Published:** 2018-07-31

**Authors:** Chuankai An, A. James O’Malley, Daniel N. Rockmore

**Affiliations:** 1 Department of Computer Science, Dartmouth College, Hanover, 03755 NH USA; 2 Department of Biomedical Data Science and the Dartmouth Institute of Health Policy and Clinical Practice in the Geisel School of Medicine at Dartmouth College, Lebanon, 03784 NH USA; 3 Department of Mathematics, Dartmouth College, Hanover, 03755 NH USA; 4 External Faculty, The Santa Fe Institute, Santa Fe, 87501 NM USA

**Keywords:** Network science, Big data, Health record analysis, Social network analysis, Predictive modeling

## Abstract

In this paper, we analyze the millions of referral paths of patients’ interactions with the healthcare system for each year in the 2006-2011 time period and relate them to U.S. cardiovascular treatment records. For a patient, a “referral path” records the chronological sequence of physicians encountered by a patient (subject to certain constraints on the times between encounters). It provides a basic unit of analysis in a broader *referral network* that encodes the flow of patients and information between physicians in a healthcare system. We consider referral networks defined over a range of interactions as well as the characteristics of referral paths, producing a characterization of the various networks as well as the physicians they comprise. We further relate these metrics and findings to outcomes in the specific area of cardiovascular care. In particular, we match a referral path to occurrences of Acute Myocardial Infarction (AMI) and use the summary measures of the referral path to predict the treatment a patient receives and medical outcomes following treatment. Some referral path features are more significant with respect to their ability to boost a tree-based predictive model, and have stronger correlations with numerical treatment outcome variables. The patterns of referral paths and the derived informative features illustrate the potential for using network science to optimize patient referrals in healthcare systems for improved treatment outcomes and more efficient utilization of medical resources.

## Introduction

A well-designed healthcare system is a crucial element of a well-functioning society, and the ways in which information and resources flow in such a system are key determinants of its efficacy. Patient referrals serve as a useful and measurable proxy for communication and collaboration between physicians in different specialties (Burns and Muller 2008). Physicians refer a patient to other physicians who will either be within or outside of their own hospital, generally (although not exclusively) for considerations relevant to the care of patients. To that end, reasons range from the need for specialized care to addressing problems of overcrowding (and thus postponing care). In aggregate, the sequence of referrals – a *referral path* – for a given patient over the course of treatment for a given concern is thus an important record of a focused interaction of a patient with the healthcare system. It represents a collection of pairwise and possibly group information sharing opportunities about treatment among two or even a team of physicians involved in the treatment of a patient.

The language (and mathematics) of network science is well-adapted to the study of such discretized and localized information and resource flow. In the particular case of healthcare we use network models and measures as a way of understanding patient care, healthcare resource allocation and treatment efficiency. To that end the referral of a patient by physician *A* to physician *B* is naturally represented as a directed edge from one network node to another. A referral path also stores the date of the visit and interactions between a patient and each physician on the path. Possibly because of specialty, different physicians might spend uneven amounts of time and effort (e.g., as measured by the relative value unit or “RVU”^1^) during a typical encounter with a patient. We describe the referral path in terms of multiple features (e.g., time between initial and final encounters or average RVU). Domains of investigation can range from the network of physicians in or attributed to a hospital, the Hospital Referral Region (HRR), or the entire United States referral network. A range of choices for edge weights can articulate different properties of these interactions. Given groups of referral network structural measures and referral path features, multilevel regression models and classification methods in machine learning have the potential to reveal relationships between the organization of patient flow in the healthcare system and the well-being of patients, and with this, insights into improving efficacy and resource allocation for our healthcare system.

In earlier work (An et al. 2018) we presented an analysis of the U.S. patient referral network, subjecting it and its HRR and state-level subnetworks to a range of network analyses to uncover their large-scale network structure. This work built on earlier work (Landon et al. 2012; Mandl et al. 2014; Lee et al. 2011; Lomi et al. 2014; Donker et al. 2010; Shea et al. 1999). Example results include the existence of power laws in degree distributions, “small-world” and core-periphery structures, and a statistical analysis of the motif structures in these networks. A suite of regressions also uncovered interesting relationships among the various network metrics. In this paper we study the more fine-scale patterns to be found in the consideration of the referral paths and importantly link these statistics to treatment outcomes in the particular setting of cardiovascular disease. While referral path and referral information generally has been ignored as a factor in the important problem of treatment outcome prediction, the predictive value of other kinds of data have been studied. In (Fiterau et al. 2017), researchers applied deep neural networks to time series of sensory data to predict other diagnoses. Several works (Liaw 2009; Ellis et al. 2008; Ball et al. 2014) in medical research mainly focused on variables from clinical medical tests and used standard statistical analysis techniques to make inferences about the relationships of treatments to outcomes.

Prior studies related to referral paths have been limited in terms of the range of health records studied (Uddin et al. 2013; Uddin 2016). In this paper we introduce new metrics related to the study of referral paths and are able to compute detailed network measures in a much larger dataset (the TDI 2 dataset) of cardiovascular disease treatment, ranging from a local hospital or HRR to the current national referral network. Aggregating the data from thousands of local hospitals and hundreds of HRRs, we use statistical methods to validate the general patterns of referral paths and referral networks. We characterize the dynamics of changes of node position and type among all physicians on a referral path. In the case of cardiovascular treatment, we find evidence of key roles on a referral path, especially for the physicians with a specialty of cardiovascular and internal medicine. We also validate the prevalence of patterns of referrals indicating that physicians work with their professional acquaintances when choosing the target of a referral, i.e., regularly send patients to the physicians who have many common collaborators. We then apply classification models to the cardiovascular referral network measures and referral path features to predict teaching status of a hospital and a patient’s treatment outcome (e.g., indicator of death within 1 year after treatment). Our considerations of networks and referral paths for cardiovascular treatment could clearly be adapted for other contexts. More specifically, given patient referral records tied to a different disease state, the metrics and methodologies we introduce here (e.g., the feature and pattern mining, model selection, analysis, etc.) could be directly adapted. In addition, our study has implications for research about a generalized notion of a referral path in such contexts as information flow in online media or social networks.

Some specific contributions of our work include:

- Novel definition of the health records-based referral path as well as novel definition of salient features for referral paths generated from both network science and time series analysis.
- Quantification of a physician’s position using centrality and other measures in the U.S. national cardiovascular referral network with the help of techniques specific to big data that are necessary for overcoming the infeasibility of using traditional algorithms for calculations at scale.
- Investigation of the patterns of millions of referral paths in the referral network, which are validated by statistical tests.
- Effective classification and regression models derived from novel referral path features and referral networks that distinguish (a) teaching status of a hospital and (b) patient treatment outcomes. These models pick up key predictors among network measures relevant to the optimization of an effective healthcare system.

## Materials, notation, and methodology

### Materials

We used Medicare beneficiary claims data for all patients diagnosed with cardiovascular disease in the U.S. during 2006-2011 to build referral paths and networks of the US healthcare system. Here cardiovascular disease means that the patient suffers from arrhythmia, congestive heart failure, coronary-heart disease or peripheral vascular disease in the diagnostic codes of Medicare claims. This dataset is of interest for several reasons. It is on the one hand a kind of network “big data” (as we will see, the data produce networks on hundreds of thousand of nodes and millions of edges) in a research area (healthcare) where traditionally data analysis has not been accomplished at this scale (i.e., related work considers data at the level of the health care unit – e.g., hospital – or a local region). In our previous work (An et al. 2018) related to national networks we had much less metadata - so that our work was more descriptive. This richer data enables us to begin to create more interesting methodologies for this kind of data. In particular, by focusing on the part of the national dataset related to a disease diagnosis, we can begin to articulate and build out methodologies that relate to outcomes. With the exception of patients dually eligible for Medicare and Medicaid, these data contain a record of each physician encounter of each Medicare patient. Each such record contains the patient or “beneficiary” (Bene) identification (ID) number, physician National Provider Identification (NPI) number, visit date, RVU associated with the visit and other details^3^. Since the NPI numbers for all physicians changed in 2007, some of the analysis we perform only obtains for the interval 2007-2011. Although claims data and other sources of patient-physician encounters has been previously used to form physician networks (An et al. 2018; Landon et al. 2012; Mandl et al. 2014; Lomi et al. 2014; Shea et al. 1999), in this paper we apply a more nuanced approach.

At the heart of this is the notion of a “referral from physician *A* to physician *B*”, which we define as the event that a patient encounters physician *B* within 30 days of encountering physician *A* (and encounters no other physician in between those times). The “referral path” is a maximal sequence of referrals, assumed to embody the team of physicians involved in the treatment of a patient over the course of a given episode of illness. A referral path might connect physicians in different areas. Since each visiting record includes the HRR and hospital where a physician is working or attributed on the basis of where most of their patients are hospitalized (Bynum et al. 2007), we can categorize referral paths as purely intra- versus inter-hospital or HRR. Similarly, the various network measures to be able to be evaluated for each HRR or hospital level (PHN) subnetwork. In this paper we will be primarily interested in *cardiovascular referral networks*, since the raw records of patient-physician visits are specific to cardiovascular disease treatment in U.S.

### Referral path

**Fig. 1:**
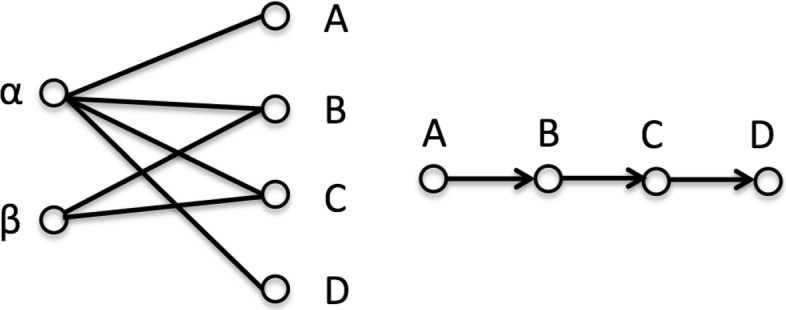
Bipartite graph between patients {*α*,*β*} and physicians {*A*,*B*,*C*,*D*}. (L) An edge between a patient and a physician means the patient visited the physician. (R) A referral path of Patient *α* in chronological order

The relationship between patients and physicians is naturally represented as a bipartite graph. In Fig. 1, several edges connect two patients (*α* and *β*) to some physicians whom they have visited. Patient *α* visits four physicians in the sequence (*A*,*B*,*C*,*D*) and patient *β* visits B and C. By sorting the four physicians according to the date of patient *α*’s visit, we recover a sequence of four physicians reflecting the sequence of encounters. In this paper, we define a patient referral path as a sequence of physicians whom the patient encounters in chronological order. If a patient encounters a physician followed by another within a threshold of 30 days (i.e., a referral exists), we assume there is an information exchange opportunity between the two physicians.

### Referral network and computation of edge weights

The *referral network* (over a given time period) is a directed network with node set given by the physicians present in the database over a fixed time period. If physician *A* refers at least one patient to physician *B*, this is represented by a directed edge from *A* to *B*. Given all referrals over a year, we are able to build the *U.S. national patient referral network of US physicians*. In this paper, we mainly investigate micro-patterns of referral paths for each patient in HRR/PHN referral networks, while our prior work (An et al. 2018) introduces macro-patterns derived from directed national, HRR, and state referral sub-networks. Herein, most of the network measures are also derived from directed referral networks, except a few measures from the corresponding undirected networks, such as diameter, clustering coefficient and giant component.

Edges can be weighted in a variety of ways. A simple unweighted edge (i.e., edge weight equal to 1) denotes simply a connection. More information is added if we use other natural metrics such as the number of referrals or the geometric mean of RVUs. A novel metric that we define here is the “ranking based weight”: Let the vector *r*=(1,2,…,*n*) denote the chronological “ranks”^4^ of the encounters on a referral path consisting of *n* physicians. In this case for a given physician *A*, let *n*_*A*_ denote the number of encounters for physician *A* on the referral path, and let *r*_*A*_ be the sub-list of the ranks of the encounters with a physician in the referral path (so, if *A* was encountered on the first and last visits only, then *r*_*A*_=(1,*n*)). In this way, *n*_*A*_ is the length of the *r*_*A*_. The flow of patients from physician *A* to physician *B* is then given by

1 $$ f_{AB} = \frac{ {\sum\nolimits}_{i<j} I\left(r_{Ai}<r_{Bj}\right) }{n_{A}n_{B}}  $$

and from *B* to *A* by

2 $$ f_{BA} = \frac{ {\sum\nolimits}_{i<j} I\left(r_{Ai}>r_{Bj}\right) }{n_{A}n_{B}}\quad.  $$

To compute the ranking based weight of an edge, we compute a weighted sum of the patient ranking index flow in each referral path *p* containing both physician *A* and *B*. A referral path *p* might include multiple physicians, but the flow of patients in the referral path between physician *A* and *B* only relates to their sub-vectors *r*_*A*_ and *r*_*B*_, without any impact from a third physician. The function of Eq. (1) lies in [0, 1], and under the assumption of a stationary model of doctor’s visit occurrence it will converge to a constant as *n*_*A*_ and *n*_*B*_ go to infinity, but we would like to account for the length of each referral path, so we add *n*_*Ap*_ and *n*_*Bp*_ and weight the contribution from each referral path by its geometric mean in Eq. (3).

3 $$ w_{AB}=\sum\limits_{p} \left(n_{Ap}n_{Bp}\right)^{1/2} f_{ABp}  $$

To sum up, Table 1 shows an interim step of the data processing process with the format of input data and the output of referral paths/networks.

**Table 1:** Example pipeline of data processing from raw patient-physician encounter records to referral paths and edges of referral network

(a) Raw visiting records		
Patient	Physician	date;HRR;HRRcity;state;zipcode;workRVU;specialty;PHN;teaching type; etc.
*α*	A	2011-01-01;1010;Hanover;NH;03755;1.0;family practice;First hospital;0;etc.
*α*	B	2011-01-10;1020;Boston;MA;02101;3.0;internal medicine;Second hospital;1; etc.
*α*	C	2011-02-01;1050;New York;NY;10021;4.0;cardiology;Third hospital;1;etc.
*β*	B	2011-03-01;1012;Lebanon;NH;03784;2.0;family practice;Fourth hospital;0;etc.
*β*	C	2011-03-20;1022;Newton;MA;02461;5.0;vascular surgery;Fifth hospital;1; etc.
(b) Referral path		
Patient		Node(date;#visiting records; RVU),divided by "->"
*α*		A(2011-01-01;1.0,1.0)->B(2011-01-10;1.0;3.0)->C(2011-02-01;1.0;4.0)
*β*		B(2011-03-01;1.0;2.0)->C(2011-03-20;1.0;5.0)
(c) Edges in the national referral network with the weights over all referral paths		
Directed edge		Weights of an edge
A->B		3; 4; 4.82; 12.14; 23.42
B->C		5; 5; 5.12; 12.32; 18.22

### Referral path features

**Fig. 2:**
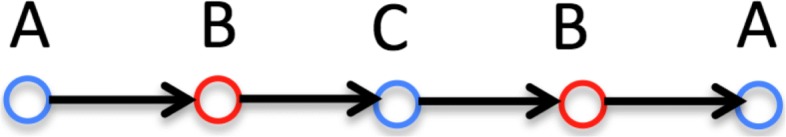
An example referral path with three physicians *A*,*B*,*C*. The patient visits them five times. Physicians *A* and *C* are from the same HRR/hospital in blue, while physician *B* is from another HRR/hospital in red

In (An et al. 2018) we introduce the use of various basic network measures for the study of patient referral networks and uncover macro-level network structures including general patterns of “power law” in degree distribution, “small-world” structure, core-periphery structure, and the existence of a “gravity law” in a state-level referral traffic map. In this paper we focus on the referral path and to that end, introduce some metrics that get at the diversity of a referral path. Denote the number of visits on a referral path as *N*, the *i*th node on a referral path as *P*_*i*_, the date of the encounter with the *i*th node as *T*_*i*_, 1≤*i*≤*N*. With this notation we make the following definitions and illustrate them using the example in Fig. 2 (note that in Fig. 2, the nodes corresponding to the physicians are color-coded according to some affiliation datum – e.g., HRR or hospital):

- *Path length*. The total number of physicians on a referral path. A physician could be counted multiple times if the patient visits the physician again. It is 5 in Fig. 2.
- *Average time gap between referrals on the referral path*: $\frac {T_{N}-T_{1}}{N-1}$.
- *Time range.* *T*_*N*_−*T*_1_. It is the gap between the last visit and the first.
- *Recurrence.* A binary variable recording whether there exists *i*,*j*, with 1≤*i*<*j*≤*N*, and *P*_*i*_=*P*_*j*_. It is true (set to “1”) in Fig. 2 because of multiple occurrences of physicians *A* and *B*.
- *Number of nodes before recurrence*. This is defined as *min*{*j*}- 1, where (*i*,*j*) satisfy the above recurrence condition. It refers to the first reappearance of a node. In our example, it is 3 since the first three nodes *A*,*B*,*C* are different from each other before the first duplicate node, *B*.
- *Physician distribution entropy.* This is the standard probabilistic definition of entropy $\left (-{\sum \nolimits }_{x}p(x)\log _{2}(x)\right)$ derived here from the physician occurrence probability over the path. In Fig. 2, the frequencies of *A*,*B*,*C* are 2,2,1 respectively. The physician distribution entropy of the related probability distribution (0.4,0.4,0.2) is 1.522.
- *Hospital distribution entropy.* The entropy of the derived physicians’ hospital distribution is another feature of diversity. Since we assume *A* and *C* are from the same hospital, the frequency distribution is (3,2) and the corresponding entropy is 0.971.
- HRR distribution entropy. The entropy of the physicians’ HRR probability is another feature of diversity. It is the same value as PHN distribution entropy under the assumption that *A* and *C* are in the same HRR.
- *Main hospital.* It is a derived referral path feature of the hospital in which the most physicians on the referral path are working. It is the hospital with *A* and *C* in Fig. 2.
- *Main or dominant HRR*. The HRR in which the most physicians are working. It is the HRR with *A* and *C* in Fig. 2.
- *Number of pairs of nodes with reciprocal referrals on a referral path.*${\sum \nolimits }_{i,j} 1\left (1\leqslant i <j \leqslant T-1, P_{i}=P_{j+1}, P_{i+1} =P_{j}\right)$. There are two pairs of nodes (*A*,*B*) and (*B*,*C*) which have such reciprocal relations.

### Node position features

In a referral network, metrics related to node characteristics correspond to metrics of physician “importance”. Meaningful examples include local clustering coefficient, betweenness centrality, closeness centrality, eigenvector centrality, PageRank centrality (Page et al. 1999), core-periphery score (Rombach et al. 2017). In addition, we adapt the notion of h-index to the patient referral network (Hirsch 2005). For a node in the national referral network, consider the array of indegrees for all nodes which refer patients to the node, then count the h-index of the indegree array, which means *h* referral source nodes have at least *h* indegree in the array.

Here are some of the features describing node position that are relevant to the context of referral paths.

- *Number of paths that contain the node*.
- *Number of paths where the node is the initial visit*. In Fig. 2, physician *A* is the first node.
- *Number of paths where the node is the final visit*. In Fig. 2, physician *A* is the end node.
- *Average index of the first-time occurrence in all paths*. In Fig. 2, the index of first-time occurrence for nodes *A*,*B*,*C* is 1,2,3, respectively, so we can take the average over all referral paths.
- *Number of paths where the node occurs multiple times*. In Fig. 2, nodes *A* and *B* occur twice.
- *Number of cross-HRR referrals proposed by the node*. In Fig. 2, given the assumption that nodes *A* and *C* are from the same HRR, node *A* sends patients to node *B* in another HRR. Nodes *B* and *C* also form an edge that spans HRRs.
- *Number of cross-hospital referrals proposed by the node*. In Fig. 2, given the assumption that nodes *A* and *C* are from the same PHN, node *A* sends patients to node *B* in another hospital. The same is true of nodes *B* and *C*.

## Results

In this project, we process raw patient-physician encounter records, build referral paths/networks and derive the following patterns in Python, with the help of NetworkX (NetworkX). We build the machine learning programs for treatment outcome prediction with scikit-learn (Scikit-learn: Machine learning in Python), and implement statistical tests and regression models in R.

### National, HRR and PHN network measures

Table 2 shows several measures (see (An et al. 2018)) over the period 2006-2011 of the big national referral network with millions of edges. Generally speaking, the national referral network restricted to U.S. cardiovascular treatment reveals macro-level patterns found in the larger “all-inclusive” U.S. referral network (An et al. 2018). For this dataset, the increased number of nodes and edges in the national referral network might be a result of the elderly population growing in number across time. We also compute those network measures for the U.S. cardiovascular treatment network restricted to the 300+ HRR and 4,800+ hospital subnetworks. In this way we characterize the standing of the physicians in a referral path nationally, locally, and institutionally. Unless otherwise noted, when we refer to a referral network in this paper we mean the US cardiovascular care patient referral network to which our methods are applied.

**Table 2:** Some national referral network measures in 2006-2011

Year	2006	2007	2008	2009	2010	2011
# nodes	272353	296008	313051	323042	334452	347586
# edges	5708791	5948185	6313136	6544847	6785594	7047586
Exponent of indegree power law	3.08	2.80	1.55	2.76	1.54	2.74
*p*-value of indegree power law test	0.97	0.89	0.21	0.85	0.22	0.82
Exponent of outdegree power law	3.01	2.69	2.71	2.66	2.56	2.68
*p*-value of outdegree power law test	0.9	0.94	0.93	0.96	0.91	0.93
Size of the largest connected component	271898	295405	312412	322452	333727	346711
(in, in) degree assortativity	-0.094	-0.088	-0.084	-0.085	-0.083	-0.084
Self in/out degree correlation	0.983	0.982	0.983	0.983	0.983	0.984
Reciprocity of #referral	0.878	0.890	0.896	0.901	0.902	0.896

### Referral path features

Table 3 describes features of millions of referral paths over 2006-2011. The average duration of each referral path is roughly 25 days (avg time range) and comprises about four nodes (avg length). About one-third of referral paths have a node which the “defining patient” visits multiple times. The distribution of the referral paths when weighted by hospital entropy is more diverse than when weighted by HRR entropy, which implies that a patient will more likely visit multiple hospitals in the same HRR than to have multiple visits in different regions (HRRs). Close to half of the pairs on a given referral path are reciprocating.

**Table 3:** Overall statistics of all referral paths in 2006-2011

Year	2006	2007	2008	2009	2010	2011
*#*referral paths	4.44M	4.45M	4.54M	4.59M	4.63M	4.66M
Avg length	3.850	3.907	3.983	4.023	4.061	4.115
Avg gap for a referral	8.509	8.506	8.369	8.352	8.230	8.060
Avg time range	24.247	24.727	24.969	25.245	25.192	25.109
Percent of paths with recurrent nodes	33.418	32.879	32.836	32.784	32.573	32.301
Avg #nodes before recurrence	4.087	4.130	4.179	4.196	4.223	4.271
Avg physician entropy	1.400	1.410	1.423	1.427	1.436	1.448
Avg hospital entropy	0.475	0.473	0.476	0.459	0.480	0.481
Avg HRR entropy	0.107	0.109	0.108	0.105	0.112	0.116
Avg bidirectional pairs in a path	0.450	0.455	0.465	0.474	0.476	0.479

### Patterns of referral paths

In addition to the basic overall features for all referral paths, we explore other patterns from other perspectives.

**Fig. 3:**
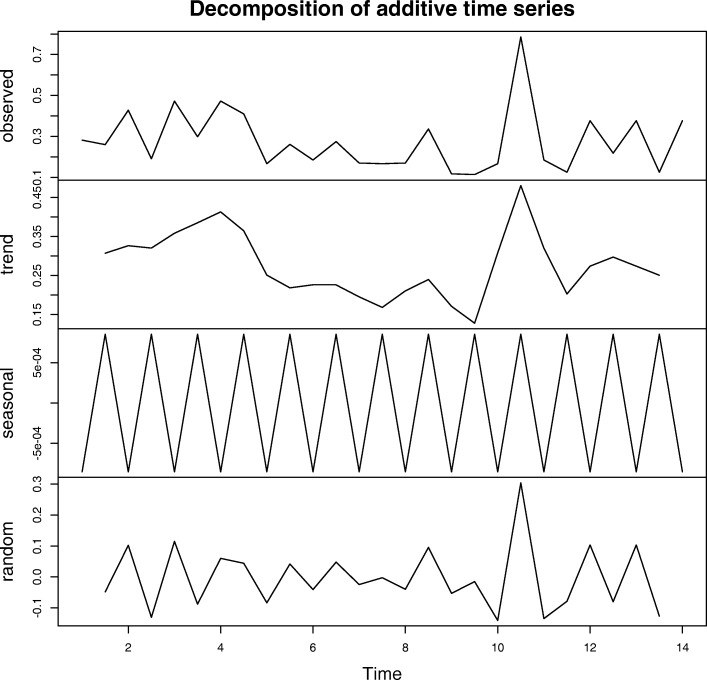
Observed local clustering coefficient of the nodes on a referral path, and the three components parsed into their time series decomposition. The seasonal component fluctuates along the time axis

**Index on Referral Path vs. Node Position in Network** Corresponding “node position sequences” encode the ways in which a patient navigates along physicians in terms of the physician position of importance in the referral network. Here we consider the node position sequence with respect to five node position measures in the national referral network: clustering coefficient, betweenness centrality, eigenvector centrality, PageRank centrality and h-index. Figure 3 shows an observed node position sequence represented by the local clustering coefficient of each node. After classical seasonal decomposition (Classical seasonal decomposition by moving averages) by moving averages on the sequence, the seasonal component tends to fluctuate, which suggests that physicians in the core and periphery parts appear alternately on the referral path.

Denote the *N* physicians on a referral path as *P*=(*P*_1_,*P*_2_,...*P*_*N*_) and the node position value of *P*_*i*_ as *C*_*i*_, so that the corresponding node position sequence can be denoted as *C*=(*C*_1_,*C*_2_,...*C*_*N*_). Then the number of changes in trend ${\sum \nolimits }_{i=2}^{N-1}1((C_{i}-C_{i-1})(C_{i+1}-C_{i})<0)$ counts the change of sgn (positive, negative) of the difference in the centrality of successive providers on referral path *P*_*i*_, 2≤*i*≤*N*−1. The event (*C*_*i*_−*C*_*i*−1_)(*C*_*i*+1_−*C*_*i*_)<0 is defined as a change point. For each node in the middle of a referral path, if the neighboring nodes and itself satisfy the condition, it contributes one to the number of change points.

Table 4 shows the percentage of change points in terms of five kinds of node position measures in 2007-2011. In most cases, a patient will alternate visits between a physician with a larger centrality measure and one with smaller centrality measure. The pattern is stable in different years with all node centrality measures, which suggests that some core physicians in the national referral network help to link some physicians with fewer referrals for the patient’s treatment.

**Table 4:** Percentage of change points in terms of increasing/decreasing trend in node position sequence of a referral path

Year	2007	2008	2009	2010	2011
Clustering coefficient	75.0	74.9	74.9	74.8	74.7
Betweenness centrality	74.9	74.7	74.8	74.7	74.5
Eigenvector centrality	74.3	74.2	74.2	74.1	74.0
PageRank centrality	74.8	74.6	74.7	74.6	74.5
h-index	70.7	70.6	70.8	70.8	70.8

The fluctuation suggests that on a referral path some physicians with relatively larger centrality measure might diagnose the disease and organize the referral path by referring the patient to nodes with lower centrality. This is the role that has been envisioned for primary care physicians in the health care system and prior network analyses (Barnett et al. 2012) have found that the more prominent (i.e., central) primary care physicians are in an intra-hospital network, the less the average cost of care at that hospital.

**Locate the key physician** Table 5 shows the top five most frequent specialties of nodes on referral paths and the top five cross-specialty referrals.

**Table 5:** Referrals to physician specialties over 2006-2011

(a) Top 5 specialties.
Cardiovascular disease
Internal medicine
Family practice
Interventional cardiology
Pulmonary disease
(b) Top 5 cross-specialty referrals.
Internal medicine → cardiovascular disease
Cardiovascular disease → internal medicine
Family practice → cardiovascular disease
Cardiovascular disease → family practice
Internal medicine → family practice

The RVU of a visit depends on the service performed or directly on the specialty of the physician. In the TDI dataset, the average RVU for physicians who specialize in cardiology, internal medicine, cardiac surgery and interventional cardiology are 4.00, 4.62, 3.65 and 3.69, respectively. The difference among specialties contributes to the uneven RVU sequence. We sort all physicians on a referral path to find the most key physician on it, allowing us to explore which kinds of physicians usually play the key role in treatment among all referral paths.

**Fig. 4:**
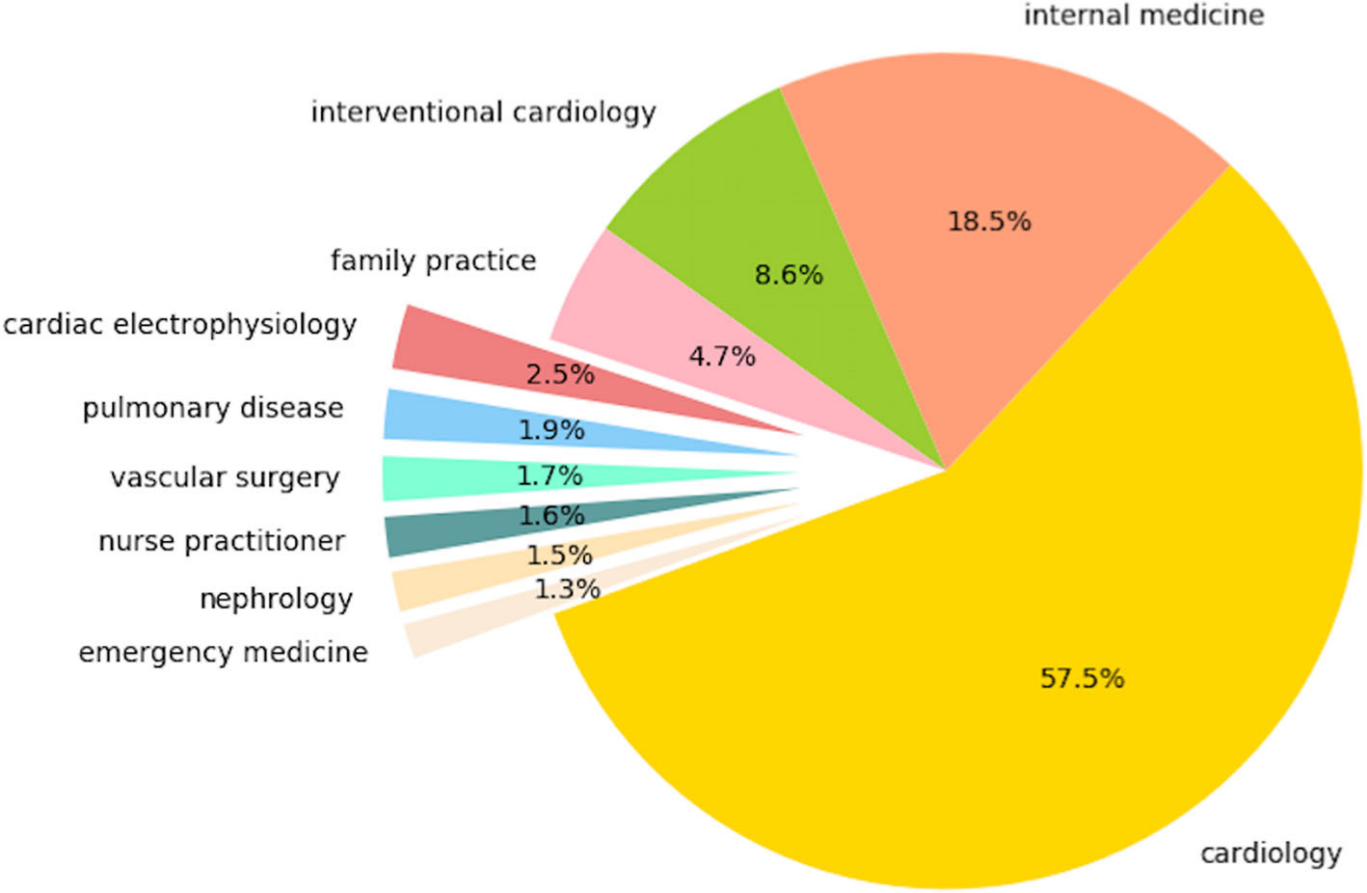
Top 10 specialties as the most key physician on referral paths in 2007-2011. Each group accounts for more than 1% of key physicians

We define a new simple metric, according to which physicians on a referral path have the smallest aggregate RVU rank and PageRank centrality rank. For example, if a physician has the largest RVU among the physicians on a referral path, and the second largest PageRank value, the sum of rank would be three. Figure 4 shows several main groups of specialties often associated with the key physician, from which we find that physicians with specialties of cardiology, internal medicine and interventional cardiology occupy a relative central position in the national referral network.

**Node position in Referral Network vs. Feature of a node on Referral Paths** Table 6 shows several strong correlations between node position measures (e.g., betweenness centrality) and referral path features. The strong correlations stem from the way we build referral networks with all referral paths. If more referral paths contain an edge, the nodes connected by the edge will have a more central position.

**Table 6:** Several pairs of strong correlations between node position and node feature on a referral path

Node position measure	Node feature about referral path	Correlation coefficient
Betweenness centrality	*#*paths with the node	0.607
PageRank centrality	*#*paths with the node	0.852
PageRank centrality	*#*paths with multiple occurrences	0.740
h-index	*#*paths with the node	0.783
h-index	*#*cross-PHN referral proposed by the physician	0.640

**Preference of collaboration** Sometimes a physician might have multiple options in terms of the target of a referral, especially when the physician is located in the center of referral networks with a wide range of connections. We compute the average number of common connected nodes for neighboring nodes in a referral path *P*, given by $\frac {{\sum \nolimits }_{i=1}^{N-1}|V(P_{i})\cap V(P_{i+1})|}{N-1}$, where *V*(*P*_*i*_) is the set of neighboring nodes of node *P*_*i*_ in the national referral network.

Table 7 shows that on average the neighbors or direct collaborators on a referral path have 25 common collaborators in the national referral network, while the expected number in a random network is $p(AX, BX | AB)=(N-2)\frac {(M-1)(M-2)}{\left (C_{2}^{N}-1\right)\left (C_{2}^{N}-2\right)}$ (*N* is the number of nodes, *M* is the number of edges). Assume there is an edge between node *A* and *B*. Then the remaining *N*−2 nodes are candidates for common neighbors. With *M*−1 edges remaining in the whole network and $C_{2}^{N}-1$ remaining pairs of possible edges, the probability that *A* and a candidate neighbor *X* are connected is $\frac {M-1}{C_{2}^{N}-1}$, which is almost the same as the ensuring conditional probability that *B* and *X* are connected. The sum of probabilities over *N*−2 candidates leads to the resulting probability being multiplied by *N*−2 to yield the expected value for the network. The clear gap in Table 7 supports a hypothesis that physicians tend to work with an acquaintance or someone in the same community when a referral is required. Among the referral steps of all referral paths in 2006-2011, only 33.2% are cross-PHN while 7.5% are cross-HRR referrals, which suggests that internal referral within the same hospital or HRR is the first choice. This suggests that actual geographic distance may be a factor for referral target selection. This would enable modeling of choice of referral targets as a ranking problem that would take into account geographic proximity (as well as possibly other factors).

**Table 7:** Comparison of average common connected nodes between neighbors on a referral path and the expectation in a random network with the same size

Year	2006	2007	2008	2009	2010	2011
Random network	3.60*E*−03	3.10*E*−03	3.00*E*−03	2.90*E*−03	2.80*E*−03	2.80*E*−03
Referral network	25.13	24.64	24.95	24.97	24.95	24.96

## Three illustrative analyses

### Teaching status classification

About 220 U.S. hospitals are members of the Council of Teaching Hospitals and Health Systems (COTH) which provide professional resources to support clinical teaching environments. In this section we show how network-level and patient referral data can be used to validate their special status as well as to better articulate the features associated with membership of COTH as well as their implications for the functioning of these hospitals. We use the network features to classify whether a hospital is a COTH member based on hospital level (PHN) referral network features. This analysis is of particular interest from a health services perspective as there is a desire to identify hospitals that act as “health care hubs” or “referral hospitals”; the hospitals that patients are referred to when solutions to their medical needs are not found elsewhere. Such hospitals are likely to be key agents in the diffusion of new medical technologies and the exnovation of outdated technologies. There is also a general interest in comparing the quality of services provided, patient outcomes, and costs of care between such referral hospitals and other hospitals. To date, a direct measure of a “referral hospital” does not exist and in lieu of that “teaching hospital” has been used as a surrogate. In this sub-section, we apply the notion of referral to posit direct measures of a referral hospital, evaluate the extent to which this correlates with COTH (Council of Teaching Hospitals) status, and evaluate the overall predictiveness of COTH status based on information in referral paths and the hospital network.

Physicians in a hospital might refer patients to other hospitals or receive patients from other hospitals. On a “traffic map”, a node represents a hospital and an edge between two nodes represents the sum of cross-hospital referrals. The in- and out-degree of a hospital are the number of other hospitals with which a hospital sends or receives patients, respectively. An intuitively appealing measure of the extent to which a hospital is a problem-solver is given by the extent to which patients are referred to that hospital from other hospitals. That is, the more patients a hospitals receives as opposed to refers, the more that it might be thought of as a “referral hospital” in this sense. The difference in referrals received minus referrals sent from a hospital is termed “net patient flow” (NPF) and is given by

$$NPF ={ \text{net patient flow}} = \# {\text{referrals in}} - \# {\text{referrals out}}.$$

We also compute the “net hospital degree” (NHD)

$$NHD = \# {\text{hospitals received patients from}} - \# {\text{hospitals send patients to}}$$

to capture the extent to which a hospital receives referrals from more hospitals than to which it sends patients.

In the health services literature (Desalegn 2013; Wu et al. 2010), COTH member hospitals have been considered referral or “go-to” hospitals. Therefore, we hypothesized that there should be a positive association between NPF/NHD and COTH status, and the higher the association, the more that the current health services definition of referral hospital is validated by these network definitions. To our knowledge, this is the first time correlates and predictors of COTH status have been examined.

NHD on the PHN traffic map alone cannot classify a hospital efficiently. In 2006-2011, of the group of hospitals with negative degree difference, 6.3*%* are teaching hospitals, while of the group of positive degree difference, 3.9*%* are teaching hospitals. Therefore, propensity to be a teaching hospital is strongly predicted by net (in minus out) degree, implying that the latter is a valid measure of the notion of a referral (or referral to hospital). We now incorporate more variables to assess if other features of the referral path or the network are associated with COTH status. Table 8 shows four groups of features of a hospital (PHN).

**Table 8:** The feature list of a hospital (PHN level referral network) for teaching hospital classification

Feature Group	Features
PHN level network measures	#nodes, #edges, gini coefficient of indegree distribution, gini coefficient of outdegree distribution, alpha of indegree power law test, alpha of outdegree power law test, diameter, global clustering coefficient, local clustering coefficient, (in, in) assortativity, self degree correlation, reciprocity of # referral, reciprocity of RVUs
Difference (in - out) of edge weights on PHN traffic map	Degree, #different referred patients, #referral, geometric mean of #visit, geometric mean of RVUs, ranking index based weight
PHN position on PHN traffic map	Local clustering coefficient, PageRank, h-index
average feature of referral paths in the PHN	Length, avg-time-gap, avg-time-range, recurrent node, # nodes before recurrence, phy-entropy, PHN-entropy, HRR-entropy, common connected nodes between neighbors, bidirectional pairs
Average node position of the PHN in the national referral network	Local clustering coefficient, PageRank, h-index

Because we have a binary classification problem on a middle-size dataset (about 4800 hospitals), we apply the following models: logistic regression (LR), K-nearest neighbors (KNN), support vector machine (SVM), decision tree (DT), random forest (RF), gradient boosting decision tree (GBDT), AdaBoost with decision trees and an equal-weighted voting method based on all the models. To reduce the risk of reverse-causality, the predictors are measured piror to the COTH label being measured, which was post 2011.

Since more than 95% of the 4800 hospitals are not teaching hospitals, only a limited number of predictors can be included in the model as there is less information with which to build the classification model than if 50% of the hospitals were not teaching hospitals. Define negative (“0”) as non-teaching status and positive (“1”) as teaching status, *tp* is true positive, *fp* is false positive, *fn* is false negative. Then we have three measures based on the confusion matrix of the accuracy of the predictions: $precision (p)= \frac {tp}{tp+fp}$$recall (r) = \frac {tp}{tp+fn}$ F-score $= \frac {2pr}{p+r}$, the harmonic mean.

To obtain a maximally interpretable model, the non-significant predictors in the above LR model and one pair of any highly collinear predictors were removed one at a time until no more predictors could be ruled out. This same procedure was applied to the interpretative versions of the predictive models of treatment and outcome following treatment and is presented later in the paper.

Table 9 shows the best two models according to the overall F-score metric. Table 10 shows some significant predictors in the Logistic Regression (LR) model with their estimated value and confidence intervals. They support the idea that referral path and referral network features matter for COTH label classification. If a feature has a positive coefficient, it means that an increase in the feature tends to make the model predict the hospital as a teaching hospital. For example, the more that referrals into the hospital exceed those departing from the hospital, the greater the NPF and the likelihood that the hospital is a COTH. Likewise, hospitals with a high h-index and long-time referral paths are more likely to be COTH hospitals. These findings make intuitive sense as it is reasonable to expect that the most complex clinical cases will on average generate the longest referral paths. However, an in-depth study that looks at medical detail beyond that captured in claims data will be needed to validate that the most severe and complex medical cases do generate the longest referral paths as opposed to an alternative explanation such as health care inefficiency.

**Table 9:** COTH classification results of Logistic Regression (LR) and Support Vector Machine (SVM)

LR	2006	2007	2008	2009	2010	2011	average F-score
Recall	0.844	0.902	0.805	0.882	0.830	0.792	
Precision	0.704	0.712	0.733	0.667	0.780	0.792	
F-score	0.768	0.796	0.767	0.759	0.804	0.792	0.781
SVM							
Recall	0.791	0.762	0.717	0.774	0.825	0.914	
Precision	0.756	0.780	0.805	0.750	0.750	0.762	
F-score	0.773	0.771	0.759	0.762	0.786	0.831	0.780

They are the best two models in terms of average F-score in 2006-2011

**Table 10:** Significant predictors in Logistic Regression for COTH classification

Feature Name	Estimated Coefficient	95% Confidence Interval	*P*-value
Gini coefficient of degree distribution in PHN network	2.823	(0.844 4.802)	5.18E-03
Global clustering coefficient of PHN network	-10.693	(-13.218 -8.167)	< 2E-16
(in, in) degree assortativity	4.813	(2.981 6.646)	2.63E-07
Difference (in-out) of # referrals on PHN traffic map	3.678	(1.630 5.726)	4.32E-04
h-index of a hospital on the PHN traffic map	7.862	(5.877 9.847)	8.37E-15
Avg time range of a referral path	5.138	(2.157 8.119)	7.29E-04
ratio of referral paths with recurrent nodes	-12.950	(-16.614 -9.286)	4.29E-12
Avg #nodes before recurrent nodes	6.139	(3.844 8.434)	1.58E-07
Avg #bidirected pairs on referral paths in the PHN	6.459	(2.407 10.512)	1.78E-03

### Patient clinical outcome and treatment received classification

We next explore whether it is possible to predict the treatment outcome for a patient based on the measures and features of the physician referral network and the referral path. Here we take a dataset of Medicare patients diagnosed with Acute Myocardial Infarction (AMI) over 2006-2011, which by virtue of the serious nature of the medical event was always diagnosed in a hospital setting. Because AMI embodies a small subset of the total claims with cardiovascular disease diagnoses, these claims are a small subset of the claims used to construct the data set of referral paths and the associated physician network. Therefore, there is no tautological dependency between the referral-path and network-based predictors based on the ensemble of cardiovascular care and the treatment outcomes of patients who experienced an AMI. The Medicare claims data record is analyzed for each patient to determine the treatments the patient received post-diagnosis and key follow-up medical events. The dataset has the following key attributes: Bene ID, admission date, death1yr (death or not within one year after index admitted date), PCI (indicator of Percutaneous Coronary Intervention within one year after index admitted date), total_payment_1yr (total real payment within one year after index admitted date). By matching the AMI admission date with the date of visit to the first physician on a referral path for the same beneficiary, we get more than 100,000 pairs of referral paths and the corresponding AMI treatment and outcome variables. If we relax the gap between the AMI admission date and the first referral path visit date to one day (as opposed to being an exact 0-day match), there will be about 22,000 more records. A further relaxation to two-days yields 3800 more records. To be cautious, we use the 0-day matching rule in the following.

The outcome death1yr and treatment PCI are both binary-valued random variables. We collect 69 kinds of features in Table 11 from referral path and patient referral network analysis, which are in six groups: network measures of the dominant HRR on the referral path, referral path features (e.g., number of nodes, time range), average node positions on the referral path, average weights of edges in the national referral network covered by the referral path, features of the last physician on the referral path (e.g., PageRank value, *#*cross-PHN referral proposed by the physician), basic patient information (e.g., age).

**Table 11:** Feature list of a referral path for treatment outcome classification/regression

Group of Features	Features and ID
Network measures in the dominant HRR	1:#nodes, 2:#edges, 3:indegree gini coefficient, 4:outdegree gini coefficient, 5:indegree power law test alpha, 6:outdegree power law test alpha, 7: diameter, 8:global clustering coefficient, 9:local clustering coefficient, 10: (in, in) assortativity, 11:self in/out degree coefficient, 12:referral reciprocity, 13:RVU reciprocity
Referral path sequence	14:#nodes, 15:average time gap, 16: time range, 17:indicator of recurrence, 18: #nodes before recurrence, 19:physician distribution entropy, 20: PHN distribution entropy, 21:HRR distribution entropy, 22:average #common connected nodes between neighbors, 23:#pairs of nodes with reciprocal referrals, 37:#change points, 38:#previous referral path in the same year, 39:distance between the first visited hospital and the end one, 40:total RVU, 41:month of the first visit, 42:#visited teaching hospitals, 43:specialty of the key physician, 44:specialty of the last physician, 45:#visited PHN with negative (in-out) degree on PHN traffic map, 46:#visited PHN with positive (in-out) degree on PHN traffic map, 47:sum of (in-out) degree for all PHN on the referral path, 60:indicator of admitted by emergency department for the first node
Average node positions on the referral path	24:local clustering coefficient, 25:PageRank, 26:h-index, 27:#paths which contains the node, 28:#paths where the node is the starting one, 29:#paths where the node is the end one, 30:index of the first-time occurrence, 31:#paths where the node occurs multiple times, 32:#cross-HRR referrals proposed by the node, 33:#cross-PHN referrals proposed by the node
Average weights of edges in the national referral network covered by the referral path	34:#referrals, 35:RVU, 36:ranking based weight
Last physician on the referral path	48:RVU, 49:month of visit, 50:local clustering coefficient, 51:PageRank, 52:h-index, 53:#paths which contains the node, 54:#paths where the node is the starting one, 55:#paths where the node is the end one, 56:average index of the first-time occurrence, 57:#paths where the node occurs multiple times, 58:#cross-HRR referrals proposed by the node, 59:#cross-PHN referrals proposed by the node
Patient history information	61:age, 62:indicator of HIV, 63:indicator of asthmatic lung disease, 64:indicator of cancer, 65:indicator of dementia, 66:indicator of diabetes, 67:indicator of liver disease, 68:indicator of chronic non-asthmatic lung disease, 69:indicator of chronic renal disease

In addition to the seven traditional classification models (LR, KNN, SVM, DT, RF, GBDT, AdaBoost), we try to boost the performance of classification with the following methods.

- **Feature engineering.** Encoding categorical attributes, such as specialty of the key physician and the month of admission date. Features are extracted using both the exact matching referral path with the AMI record and the immediately preceding referral path within the 90 day period before the exact matching one, in order to capture the association between referral path features and subsequent treatment outcomes.
- **10-fold cross validations**. Accomplished by partitioning the original sample into a training set and a test set in rotation.
- **Undersampling.** Undersample some training cases to balance the ratio of positive/negative in training set.
- **Feature selection.** Apply Random Forest (RF) to sort features by their importance (Genuer et al. 2010), and pick up a subset of important features for classification models. Here the importance of a given feature is the increase in mean error of a tree in the forest when the observed values of this feature are randomly permuted.
- **Voting for the final label.** Collect prediction result of each classification model and vote for the final prediction result of a test case.
- **Xgboost (**Chen and Guestrin 2016**).** Upgrade the gradient boosting model from GBDT to Xgboost, which aims to strengthen regularization of trees and control overfitting.

GBDT has the highest F-score with its performance depicted in Table 12 for each year and outcome. Since we can tune parameters in a classification model to get a higher recall or precision, the F-score is more meaningful as an overall evaluation metric. The moderate F-score suggests that a lot of unmeasured variables contribute to treatment decisions and patients’ survival. The lack of clinical detail and personal information such as heart rate and blood pressure weakens the power of machine learning models, but the referral path features and network measures support the above models to beat random prediction while the accuracy is almost as good as that of other diagnosis classification. A complex convolutional neural network (CNN) model (Fiterau et al. 2017) aims to predict osteoarthritis with much more (600+) directly related features (e.g., clinical measures, joint symptoms/function) and 7-day time series accelerometer sensory data, but the accuracy of baselines and the CNN ranges from 0.633 to 0.789. Table 13 shows the average F-score for death1yr and PCI classification on two separate groups divided by age. The power of referral path features differs, which means age is an important factor. As predictability does not necessarily imply causality, to attain rigorous causal inferences to the standard typical in medical research would require more study regarding potential confounding variables and possibly involve a randomized study. Moreover, if available, we should group by referral paths based on clinical tests and demographics, because it will be clear to see the effects of referral paths among a group of similar patients before treatment.

**Table 12:** Classification results of GBDT for death1yr and PCI in 2007-2011

PCI	2007	2008	2009	2010	2011	Average F-score
Recall	0.703	0.700	0.702	0.695	0.694	
Precision	0.572	0.574	0.585	0.597	0.607	
F-score	0.631	0.630	0.638	0.642	0.647	0.638
death1yr						
Recall	0.702	0.698	0.710	0.704	0.682	
Precision	0.640	0.632	0.639	0.650	0.633	
F-score	0.669	0.663	0.672	0.675	0.657	0.667

**Table 13:** Average F-score in 2007-2011 of GBDT on groups divided by age

	Death1yr	PCI
Age<=75	0.592	0.695
Age>75	0.687	0.565

Table 14 shows the top 10 important features for two indicators in 2011, which are selected by the result of RF (Genuer et al. 2010). For both death1yr and PCI, average time gap on the referral path is one of the most important features. We conjecture that the gap reflects whether the case is serious. In addition, total RVU of physicians on the referral path is predictive of death1yr (the patient outcome) and physician position (measured by PageRank) is predictive of PCI (the patient treatment received). Moreover, Table 15 contains some significant predictors in the logistic regression (LR) model for the two binary indicators. The above significant features offer new directions for medical researchers to investigate with their domain knowledge.

**Table 14:** Top 10 important features for death1yr and PCI generated by Random Forest feature selection method (Genuer et al. 2010)

Rank	Death1yr	PCI
1	Total RVU of the referral path	Average time gap on the referral path
2	Total RVU of the previous referral path	Indicator of patient’s age in 66-70
3	Average time gap on the referral path	Average PageRank values of all physicians on the referral path
4	Time range of the referral path	Indicator of the key physician’s specialty on the referral path as “interventional cardiology”
5	Average index of the first-time occurrence on a referral path for the last physician	Indicator of patient’s age in 76+
6	Local clustering coefficient of the last physician on the referral path	The number of referral paths that include the last physician
7	Times of being the end node on a referral path of the last physician on the referral path	Indicator of the key physician’s specialty on the referral path as “interventional cardiology”
8	Times of being the first node on a referral path for the last physician	Average #involved paths among physicians on the referral path
9	indicator of patient’s age in 76+	Average times of being the first node on a referral path for all physicians on the referral path
10	Average times of being the end node on a referral path for all physicians on the referral path	Times of being the first node on a referral path for the last physician

**Table 15:** Significant predictors for LR on two binary treatment variables with estimated coefficients in 95% confidence interval (CI)

(a) death1yr			
Feature	Estimate	95*%* CI	*p*-value
#nodes in domain HRR	−0.243	(−0.389−0.098)	1.03*E*−03
Physician distribution entropy	−0.313	(−0.625−0.0013)	0.049
PHN distribution entropy	−0.528	(−0.692−0.365)	2.34*E*−10
#pairs of nodes with reciprocal referrals	−2.496	(−3.666−1.325)	2.93*E*−05
Avg. PageRank values on a referral path	−2.290	(−2.803−1.778)	<2*E*−16
Avg. index of first occurrence	−0.569	(−0.974−0.164)	0.0059
Avg. proposed #cross-PHN referrals	1.628	(0.961 2.295)	1.73*E*−06
Avg. #referrals on the corresponding edges	8.696	(4.771 12.620)	1.41*E*−05
Avg. ranking-based weight on the corresponding edges	−3.973	(−6.426−1.519)	0.0015
#previous paths	2.204	(1.908 2.500)	<2*E*−16
Total RVU	11.414	(10.461 12.367)	<2*E*−16
Times of being the end node of the last physician	−2.985	(−4.075−1.896)	7.89*E*−08
Avg. first occurrence index of the last physician	4.869	(4.176 5.562)	<2*E*−16
Times of occurring multiple times of the last physician	1.778	(1.041 2.514)	2.23*E*−06
(b) PCI			
Feature	Estimate	95% CI	*p*-value
Physician distribution entropy	−0.368	(−0.678−0.058)	0.019
PHN distribution entropy	0.547	(0.359 0.734)	1.08*E*−08
Avg. #common connected nodes between neighbors	0.487	(0.097 0.877)	0.014
Avg. PageRank values on a referral path	3.874	(3.337 4.411)	<2*E*−16
Avg. proposed #cross-PHN referrals	−1.738	(−2.278−1.197)	2.89*E*−10
Avg. #referrals on the corresponding edges	−2.222	(−3.822−0.622)	0.0065
#previous paths	−1.845	(−2.155−1.533)	<2*E*−16
Total RVU	−2.113	(−2.909−1.315)	2.02*E*−07
Local clustering coefficient of the last physician	−1.352	(−1.969−0.735)	1.76*E*−05
Avg. first occurrence index of the last physician	−3.024	(−4.034−2.013)	4.48*E*−09

GBDT’s level of predictive accuracy was on average higher than LR for predicting PCI and higher than LR for predicting death within a year. However, the form of the model from LR is the most amenable to interpreting the model and determining which terms are the most predictive. For this reason, Table 15 shows the estimated parameter values, 95% confidence interval limits, and *p*-values for the PCI treatment selection and the 1-year death models. The direction of the estimated coefficients might be helpful to reveal some important relationships. For example, for Feature 34 (weights of edges covered by the referral path) in the dealth1yr model, the estimated coefficient implies that referrals of a patient to core physicians in the referral network are associated with death within the year. For Feature 25 (average PageRank value of physicians on a referral path) in the PCI model, the positive coefficient suggests that core physicians tend to treat the patients who are most likely to undergo PCI.

### Linear regression analysis of log(total 1yr payments)

In addition to categorical treatment outcome variables, we also use regression to explore the relationship between referral path features and total_payment_1yr. A feature of Medicare claims data is that any patient in the dataset must have had at least one encounter with a physician in order to enter the dataset. Therefore, their annual cost of care will be non-zero. However, cost data is notorious for exhibiting right skew. Therefore, we model log(Total Cost) as opposed to Total Cost itself using a linear regression model.

4 $$  \log(Y_{t}) = \lambda_{t} + \boldsymbol{\beta}_{1}^{T} \boldsymbol{X}_{t}+\boldsymbol{\beta}_{2}^{T} \boldsymbol{X}_{t}t + \varepsilon_{t}  $$

The form of the model is given in Eq. 4, where *Y*_*t*_ and *X*_*t*_ are dependent treatment outcome variables (e.g., total 1yr payments) and the vector of referral path features, respectively, with the outcomes measured over 2007–2011. The network related measures in *X*_*t*_, which are only measured once per calendar year, are lagged in the years of 2006–2010 to make sure that *Y*_*t*_ is measured after *X*_*t*_.

The main effect of the vector of referral path features is *β*_1_ while its modification by year *t* is *β*_2_, although in our primary analysis we focus on the model in which *β*_2_=0. The parameters *λ*_*t*_ allow for an unstructured trend across time, and $\varepsilon _{t} \sim N\left (0,\sigma _{log(y)}^{2}\right)$ describes the distribution of the error term. Modeling time in 2007–2011 categorically as a main effect and linearly as a modifier of referral path features serves the purpose of allowing a maximally flexible trend.

The fitted models appear to have a high level of face validity. For example, higher RVU is associated with greater total cost of treatment. Other significant predictors of log(total_payment_1yr), their estimated coefficients ***β***_1_, 95% confidence intervals, and *p*-values are also shown in Table 16. The asterisk represents significant interactions with time, which is assessed by estimating the full model in (4).

**Table 16:** Significant predictors in multiple linear regression models for log(Total 1yr payments) with estimated coefficients in %95 confidence interval (CI)

Feature	Estimate	CI	*p*-value
#nodes in domain HRR	0.121	(0.099 0.142)	< 2*E*−16
referral reciprocity in domain HRR	0.209	(0.167 0.251)	< 2*E*−16
#nodes ^∗^	- 2.588	(−2.992−2.183)	< 2*E*−16
Physician distribution entropy	1.365	(1.321 1.410)	< 2*E*−16
PHN distribution entropy ^∗^	0.413	(0.347 0.480)	< 2*E*−16
Avg. #common connected nodes between neighbors	− 0.357	(−0.432−0.282)	< 2*E*−16
#pairs of nodes with reciprocal referrals	2.618	(2.374 2.863)	< 2*E*−16
Avg. local clustering coefficient on the referral path	− 1.222	(−1.326−1.117)	< 2*E*−16
Avg. PageRank values on the referral path	0.983	(0.888 1.077)	< 2*E*−16
Avg. index of first occurrence on the referral path	0.341	(0.235 0.447)	3.05*E*−10
Avg. proposed #cross-PHN referrals	− 0.592	(−0.685−0.498)	< 2*E*−16
Avg. #referrals on the corresponding edges	−0.567	(−0.902−0.232)	9.25*E*−04
Avg. ranking-based weight on the corresponding edges ^∗^	0.775	(0.485 1.064)	1.59*E*−07
#previous paths ^∗^	0.304	(0.212 0.396)	9.28*E*−11
Total RVU ^∗^	5.028	(4.604 5.451)	< 2*E*−16
Month of the first visit	categorical	vary for groups	< 2*E*−16
Specialty of the key physician	categorical	vary for groups	< 2*E*−16
Month of the last visit	categorical	vary for groups	< 2*E*−16
Avg. first occurrence index of the last physician ^∗^	− 0.433	(−0.686−0.179)	7.99*E*−04

Asterisk means the predictor has significant interactions with time

## In-depth study of a hospital

**Fig. 5:**
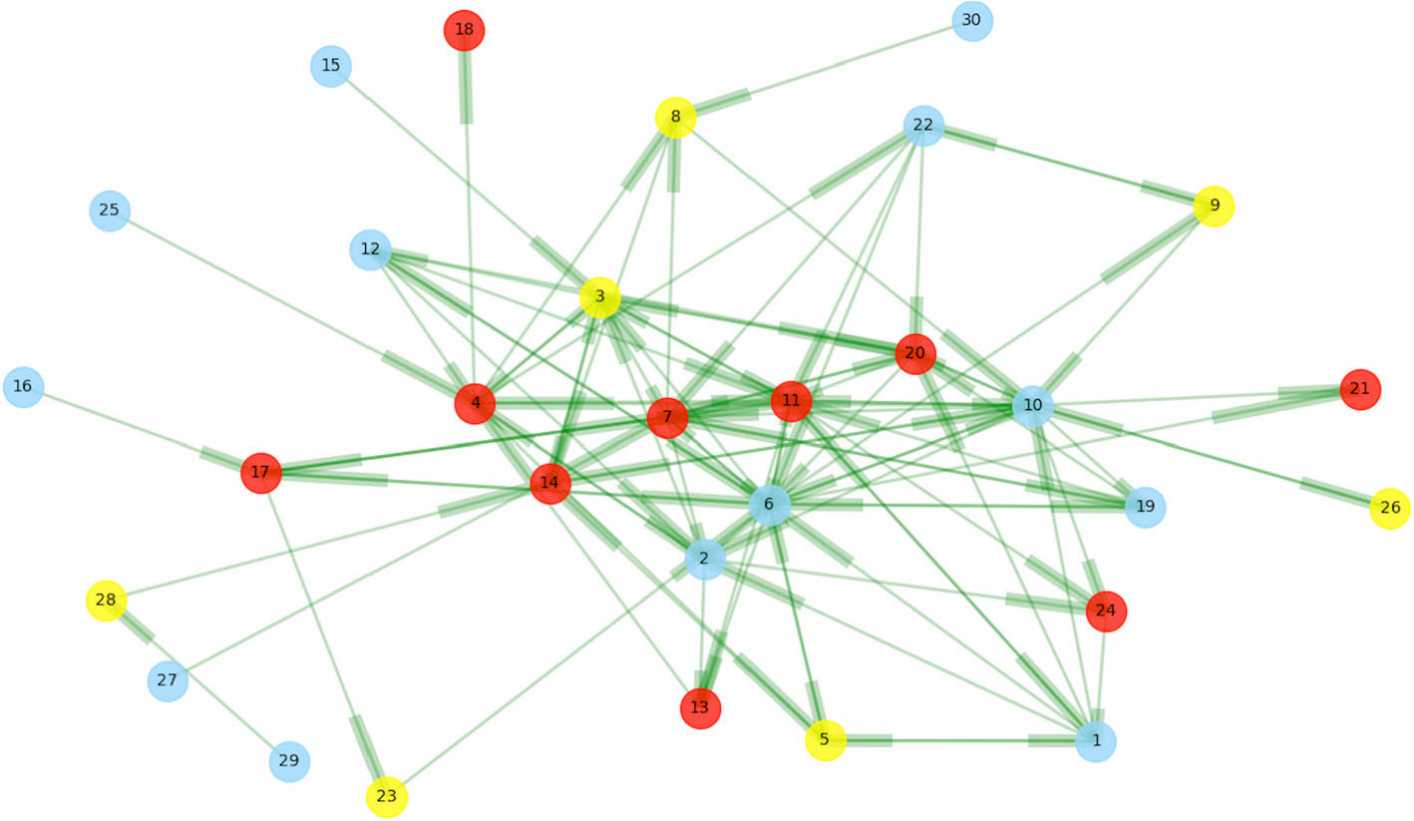
Visualization of a hospital (PHN) referral network with 30 physicians and 101 directed edges in 2011. Red, yellow and lightblue nodes represent physicians with positive, zero and negative net patient flow (NPF), respectively. Targets of referrals are marked with shadow on directed edges. The edge weights are in Table 17

Figure 5 shows a PHN level patient referral network. Table 17 shows the weights of some directed edges. The hospital is a non-teaching hospital, the Mark Twain Medical Center (in CA), with national provider ID 051332. The *p*-values of the indegree and outdegree distribution power law test are 0.21 and 0.73, respectively. The global clustering coefficient is 0.37 and the local clustering is 0.35. The average PageRank value of all nodes in the national referral network is 1.71×10^−6^. On the hospital traffic map, the net hospital degree (NHD) is 3 and the NPF is − 43.

**Table 17:** A part of network weights in the PHN network of Fig. 5

(s, t, w)	(s, t, w)	(s, t, w)	(s, t, w)	(s, t, w)
(20, 7, 2)	(1, 6, 2)	(22, 6, 2)	(6, 7, 11)	(10, 7, 2)
(7, 6, 10)	(6, 10, 3)	(7, 19, 2)	(3, 12, 2)	(12, 2, 2)
(17, 6, 2)	(17, 11, 3)	(6, 11, 4)	(7, 17, 3)	(14, 3, 2)
(6, 17, 5)	(8, 10, 2)	(14, 10, 2)	(19, 6, 3)	(4, 2, 2)
(2, 4, 4)	(10, 11, 3)	(11, 24, 2)	(3, 14, 2)	(11, 6, 2)

The weights (i.e. number of referrals) of the remaining edges are one. A triple means (source, target, weight)

Some nodes have more connections with others in the hospital (PHN), such as physician 6. The PageRank value of physician 6 is 5.36×10^−6^ while that of the physician 15, who is on the periphery, is 2.07×10^−6^. In the 2011 national referral network, physician 6 is the first/end node in a referral path 38/26 times among 75 occurrences in total. The average index of the first occurrence in a referral path is 2.03. Physician 6 initiates 24 cross-HRR referrals and 49 cross-PHN referrals.

The following are some overall features based on all referral paths whose dominant HRR is the hospital. The average length is 3.10, average time range is 25.45 (days), 30% of referral paths have recurrent nodes, each referral path has 0.37 pairs of bidirected nodes, 3.67 nodes are before the recurrent nodes, entropy of physician distribution is 1.257, entropy of hospital distribution is 0.71, entropy of HRR distribution is 0.55, the neighboring nodes on referral paths have 7.2 common connected neighbors in the national referral network. The above referral path-related features are applied to predict the COTH label of the hospital.

The following is an example of a referral path: a patient visited Node 19, Node 6, an external Node E1 (some node in another hospital), Node 6, Node E1, Node 17 within 45 days. For the neighboring nodes on the referral path, on average they had 7.8 nodes (physicians) in common connection in 2011. The total RVU during the period of referrals was 20.52. No teaching hospital provided treatment to the patient. Four of the six physicians worked in a hospital (PHN) with negative NHD. Among 31 PHNs which had connections with the PHN 051332 in Fig. 5, PHN 050084 St. Joseph’s Medical Center in CA, sent and received the most patients to and from PHN 051332. The straight-line distance between the two hospitals is only 59km. Since they are located in the vast rural area of CA, their relationship reflects the “gravity law” described in (An et al. 2018).

## Conclusions

In this paper, we apply algorithms and models from network science, statistics and machine learning to define the notion of the referral path and to derive features of interest to explore patterns in the U.S.-based cardiovascular patient referral networks. Firstly, since referral path features mainly describe micro-patterns related to patient referrals, a better understanding of referral features may provide insights into directions for improving the healthcare system. For example, node position values on a referral path change frequently in terms of increasing/decreasing trend, so we realize the significant role of physicians with relative larger centrality measures in referral networks when they build connections in the network. Physicians could also identify their position in the large community of physicians and set a goal of collaborations for career development. Moreover, physicians tend to send a patient to another physician who has a lot of common connected neighbors in the national referral network, suggesting that network structural position of physicians is a marker of their reputation and prominence among their colleagues. Our pattern-mining processing of the millions of cardiovascular disease treatment records and subsequent network, statistical and big data analysis can readily be applied to other diseases. Ultimately, we hope to develop a network science toolkit (measures, tools, and models) available to medical researchers for use in their own research.

Second, we explored applications of the referral paths features and referral network measures on teaching hospital (COTH) classification for 4,800 hospitals in the U.S. The fact that the referral-path definitions of a “referral” or “hub” hospital based on differences of patient flows into and from a hospital were strong predictors but far from perfect predictors of COTH status is encouraging vis-a-vis the utility of these new measures. On the one hand, it provides a form of face validity of the referral-path based measures; if they had no predictive power we would doubt their validity. However, by being far from perfect predictors of COTH status, this leaves open the strong possibility that a much more informative measure or indicator of a referral (problem-solving) hospital can be constructed. Such a measure will aid health services researchers and other researchers interested in the structure and consequences of the U.S. healthcare system. We believe hospitals could learn from the patterns and results derived herein in the following ways: when a successful new treatment is approved, identifying the network positions or features of the first physicians and hospitals to adopt may help to enable such influential physicians to be identified as well as provide network-based insights into the keys underlying their success. Those non-teaching hospitals that are most like teaching hospitals in their network characteristics might be the best candidates to become teaching hospitals. Analogously, those teaching hospitals that are the most like non-teaching hospitals might be in need to re-structuring.

Third, by linking AMI treatment and outcome variables to the corresponding referral paths, we find several informative predictors with either larger feature importance or significant effects, such as the time gap between two visits on the referral path and the total RVUs of all physicians’ endeavors. The novelty of these referral path measures suggests that a deeper look into their significance is warranted. We have only just scratched the surface of the enormous potential for using referral path features to improve predictions of treatment received and treatment outcomes. Understanding referral path patterns has the potential to ultimately help hospitals, physicians and patients towards the ultimate goal of building an optimal referral path for each patient with a better treatment outcome and providing the most effective allocation of medical resources.

## Abbreviations

AMI: Acute myocardial infarction
CI: Confidence interval
CNN: Convolutional neural network
COTH: Council of teaching hospitals and health systems
DT: Decision tree
GBDT: Gradient boosting decision tree
HRR(s): Hospital referral region(s)
KNN: K-nearest neighbors
LR: Logistic regression
NHD: Net hospital degree
NPF: Net patient flow
NPI: National provider identification
PCI: Percutaneous coronary intervention
PHN: Referral network in hospital level
RF: Random forest
RVU: Relative value unit
SVM: Support vector machine
TDI: The dartmouth institute for health policy and clinical practice

## Footnotes

1 RVU stands for “relative value unit”. This is a Medicare invention used in the calculation of reimbursements that encodes the “value” of a given procedure.

2 The Dartmouth Institute for Health Policy and Clinical Practice

3 Example fields include city, HRR, state, zip code and teaching type of the hospital, the specialty of the physician, etc.

4 The list of positions – denoting first, second,...,*n*th – in the sequence of *n* visits that make up the referral path.
